# Distinct Visual Working Memory Systems for View-Dependent and View-Invariant Representation

**DOI:** 10.1371/journal.pone.0006601

**Published:** 2009-08-11

**Authors:** Justin N. Wood

**Affiliations:** Department of Psychology, University of Southern California, Los Angeles, California, United States of America; University of Sydney, Australia

## Abstract

**Background:**

How do people sustain a visual representation of the environment? Currently, many researchers argue that a single visual working memory system sustains non-spatial object information such as colors and shapes. However, previous studies tested visual working memory for two-dimensional objects only. In consequence, the nature of visual working memory for three-dimensional (3D) object representation remains unknown.

**Methodology/Principal Findings:**

Here, I show that when sustaining information about 3D objects, visual working memory clearly divides into two separate, specialized memory systems, rather than one system, as was previously thought. One memory system gradually accumulates sensory information, forming an increasingly precise view-dependent representation of the scene over the course of several seconds. A second memory system sustains view-invariant representations of 3D objects. The view-dependent memory system has a storage capacity of 3–4 representations and the view-invariant memory system has a storage capacity of 1–2 representations. These systems can operate independently from one another and do not compete for working memory storage resources.

**Conclusions/Significance:**

These results provide evidence that visual working memory sustains object information in two separate, specialized memory systems. One memory system sustains view-dependent representations of the scene, akin to the view-specific representations that guide place recognition during navigation in humans, rodents and insects. The second memory system sustains view-invariant representations of 3D objects, akin to the object-based representations that underlie object cognition.

## Introduction

Cognitive abilities use both view-dependent sensory representations of the scene and view-invariant representations of individual objects. View-dependent representations support the primary mechanism of place recognition in animals: a view-matching ‘snapshot’ system. In brief, an animal takes a visual ‘snapshot’ of the scene surrounding a target goal (e.g., a nest) and stores this view in memory. During navigation, the animal moves in order to recover this target view so as to reduce the difference between the current view and the target view [1], [2].

Evidence for snapshot representations comes from studies of navigating insects and mammals. Bees, for example, were trained to forage in an environment filled with landmarks and then the locations of the food source and the landmarks were moved. Bees approached the food source from a constant direction, so that the visual image of the scene was roughly the same each time they approached the food [3], [4]. Some insects such as wood ants store multiple snapshots of a familiar landmark from different vantage points so that they may approach a familiar landmark from multiple angles [5]. Snapshot representations also guide place recognition during navigation in rodents and humans [6]–[9]. For instance, rodents in a water maze approach a hidden support from a familiar direction [6], which suggests that they use view-specific representations to recognize their location in the maze. Further, for human adults, place recognition during navigation can be based solely on smooth variations of color and intensity in the visual snapshot, in qualitative agreement with a view-matching snapshot system but not with other models of place recognition [7].

A snapshot representation consists of a relatively unprocessed sensory representation of the scene [10]. Thus, the information stored about an object in a snapshot representation is limited to the sensory features in the visual image. This differs significantly from view-invariant representations of three-dimensional (3D) objects, which include information about conceptual properties of objects. In particular, when we recognize a 3D object we not only establish a match to a stored object representation, but we also access a conceptually meaningful representation of the object's kind or a rich representation of the particular object [11]. This provides a wealth of information about that object, such as its function, perceived 3D shape, and so on. View-invariant representations of 3D objects are therefore the input for the many perceptual and cognitive abilities that operate over representations of individual objects – for example, we categorize objects, reach for objects, imagine objects, count objects, and represent the causal interactions of objects.

How do people sustain view-dependent and view-invariant representations? Both types of representation need to be sustained in a temporary memory buffer, known as visual working memory [12], in order to guide behavior. View-dependent processes require visual working memory to sustain information across eye movements, to build a stable view-specific representation that characterizes one's location in the environment. View-invariant processes require visual working memory to sustain information about the identities of individual objects across visual interruptions, such as when objects are occluded by other objects.

However, it is unclear how visual working memory sustains view-dependent and view-invariant representations. Visual working memory is widely thought to consist of a single system for sustaining visual shapes and colors, with a storage capacity of 3–4 integrated object representations [13]–[16]. Thus, under such ‘single-system’ models, the same visual working memory system sustains both view-dependent and view-invariant representations. Crucially, this poses a significant challenge for single-system models because view-dependent and view-invariant representations require different types of memory mechanisms. View-dependent representations depend on a memory system that sustains relatively unprocessed sensory information from the scene [17], whereas view-invariant representations depend on a memory system that sustains information about 3D objects independent of the particular sensory features produced by the objects [18]. To solve this problem, the visual system may have evolved separate, specialized memory systems to sustain view-dependent and view-invariant representations. The current study explores this possibility by testing several unique predictions generated by this ‘two-system’ hypothesis.

## Results

### Experiment 1

**Figure 1:**
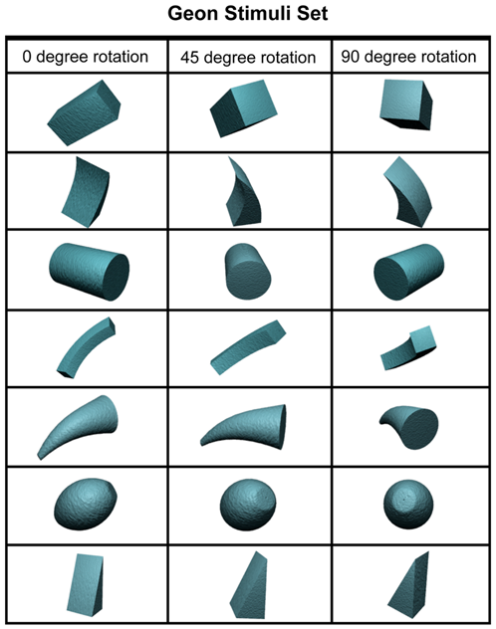
Geon Stimuli Set used in Experiments 1–3. Depiction of each of the seven geons from the three different viewpoints. Images courtesy of Michael J. Tarr, Brown University, http://www.tarrlab.org/.

**Figure 2:**
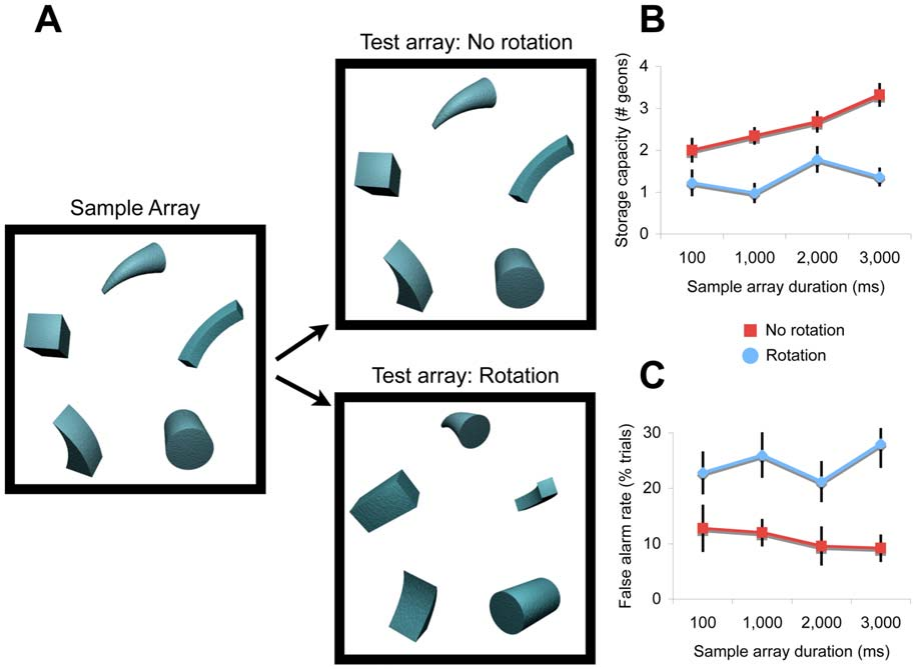
Design and Results from Experiment 1. (A) Observers viewed a sample array and a test array on each trial, separated by a 1,000-ms delay interval, and then indicated whether the same objects were present in both arrays. In separate conditions, the sample arrays were displayed for 100 ms, 1,000 ms, 2,000 ms, and 3,000 ms. In the ‘no rotation’ condition, the objects were presented from the same viewpoints in the sample and test arrays; in the ‘rotation’ condition, the objects were presented from different viewpoints in the arrays (45° or 90° rotation). (B) The storage capacity of visual working memory for non-rotated and rotated objects as a function of the display time of the sample arrays. Error bars represent standard error. (C) Percentage of false alarms for non-rotated and rotated objects as a function of the display time of the sample arrays. Error bars represent standard error.

To examine the informational content of the representations sustained in visual working memory, I used the sequential comparison procedure [19]. Observers (*n* = 10) viewed a sample array and a test array on each trial, separated by a brief delay, and then indicated whether the two arrays were identical or differed in terms of a single object. Each array contained five “geon” objects, arguably the most basic units of 3D object representation [18] (see geon stimuli set, Figure 1). In the ‘no rotation’ condition, the geons were presented from the same viewpoints in the sample and test arrays. In the ‘rotation’ condition, the geons were presented from different but similarly recognizable viewpoints in the sample and test arrays (see Figure 2A). When objects are observed from different viewpoints, they produce different low-level sensory features in the visual image [18]. Thus, view-dependent representations, which consist of low-level sensory features from the visual image [10], were sufficient to detect a geon change in the ‘no rotation’ condition, but were not sufficient to detect a geon change in the ‘rotation’ condition. The sample arrays were presented for varying durations, ranging from 100 ms to 3,000 ms. During all trials observers performed an articulatory suppression task to rule out verbal contamination of visual working memory capacity [20].

For the statistical analyses, the data were converted into capacity estimates by using the formula, *k* = *n*×(*H*–*F*), developed by Cowan [21]. If an observer can retain *k* items from an array consisting of *n* items, then the observer should be able to detect a change in one of the items on *k*/*n* trials. This approach considers the effects of guessing, by factoring in the false alarm rate, *F* = false alarms/(false alarms+correct rejections) and the observed hit rate, *H* = hits/(hits+misses). The number of items or geons, *k*, that can be retained in the ‘rotation’ condition therefore provides an estimate of the storage capacity of visual working memory for 3D object representation.

Results show that the storage capacity of visual working memory was substantially higher in the ‘no rotation’ condition compared to the ‘rotation’ condition, *F*(1,9) = 67.78, *P*<.0001 (Figure 2B; Table 1). In the ‘rotation’ condition, when view-invariant representations were needed to remember the geons, observers remembered a maximum of 1–2 geons, whether the sample array was displayed for 100 ms or 3,000 ms, *F*(1,9) = 0.25, *P* = .63. The fact that memory capacity was not significantly influenced by variations in the duration of the sample array indicates that performance was not limited by processes other than working memory storage, such as for perceiving the stimuli and encoding the stimuli into memory. Conversely, in the ‘no rotation’ condition, when view-dependent representations could also be used to remember the geons, capacity was much higher and increased monotonically along with increases in the duration of the sample array, *F*(3,27) = 6.71, *P* = .002 (see Figure 2B). This indicates that observers gradually accumulated sensory information from the sample arrays, forming increasingly precise view-specific representations in visual working memory over the course of several seconds.

**Table 1:** Proportions of Hits and False Alarms (FAs) for Experiment 1 (Hits/FAs).

Sample duration	‘No rotation’ condition	‘Rotation’ condition
**100 ms**	.50/.13	.43/.23
**1,000 ms**	.59/.12	.45/.26
**2,000 ms**	.63/.10	.57/.21
**3,000 ms**	.76/.09	.55/.28

Includes proportion of Hits (responding *different* on change trials) and False Alarms (responding *different* on same trials) for each of the conditions.

Memory capacity could have been lower in the ‘rotation’ condition either because view-invariant representations require more storage resources from a single visual working memory system than view-dependent representations (i.e., 1–2 view-invariant representations was sufficient to deplete the 3–4 discrete, fixed resolution storage slots of visual working memory [22], [23]) or because only the view-invariant memory system, which has a storage capacity of 1–2 representations, can sustain representations of 3D objects.

However, if observers used two parallel visual working memory systems, then both systems should have sustained visual information from the sample arrays. Thus, despite performing a view-invariant memory task in the ‘rotation’ condition, observers should nonetheless have also sustained view-dependent sensory representations. This would have caused the view-dependent representations to mismatch with the sensory properties of the rotated objects in the test array, creating the perception that a geon was replaced with a new geon on a large proportion of the trials. Results support this parallel storage prediction. Observers were more likely to false alarm in the ‘rotation’ condition than in the ‘no rotation’ condition, *F*(1,9) = 19.30, *P* = .002 (Figure 2C), which suggests that observers use both view-dependent and view-invariant representations in parallel to represent the visual environment. Both types of representation are durable and functional and support an observer's perception of a stable world.

Experiment 1 provides three kinds of evidence that distinct memory systems sustain view-dependent and view-invariant representations. First, view-dependent memory and view-invariant memory have different storage capacity limits. Second, view-dependent memory, but not view-invariant memory, continues to accumulate information over the course of several seconds from a scene of 3D objects. Third, memory for 3D objects presented from different viewpoints in sample and test arrays produces a different pattern of errors than memory for objects presented from the same viewpoints in the arrays. There may, however, be alternative explanations that can account for the data. The remaining experiments were conducted to ensure that performance truly reflected the capacities of two separate, specialized visual working memory systems and was not influenced by verbal working memory or by limitations in retrieval processes.

### Experiment 2

One of the primary empirical criteria for separate working memory systems is that each system is subject to its own information storage limit. Thus, it should be possible to sustain information in two separate working memory systems in parallel with little to no competition between the systems for limited storage resources. The most common method for measuring whether two types of information can be stored in separate working memory systems is the dual-task method, in which participants are asked to perform two working memory tasks concurrently. If one memory task disrupts the other memory task, then the two tasks may use the same working memory system. If performance on the two memory tasks are independent of one another, then the two tasks use separate working memory systems.

**Figure 3:**
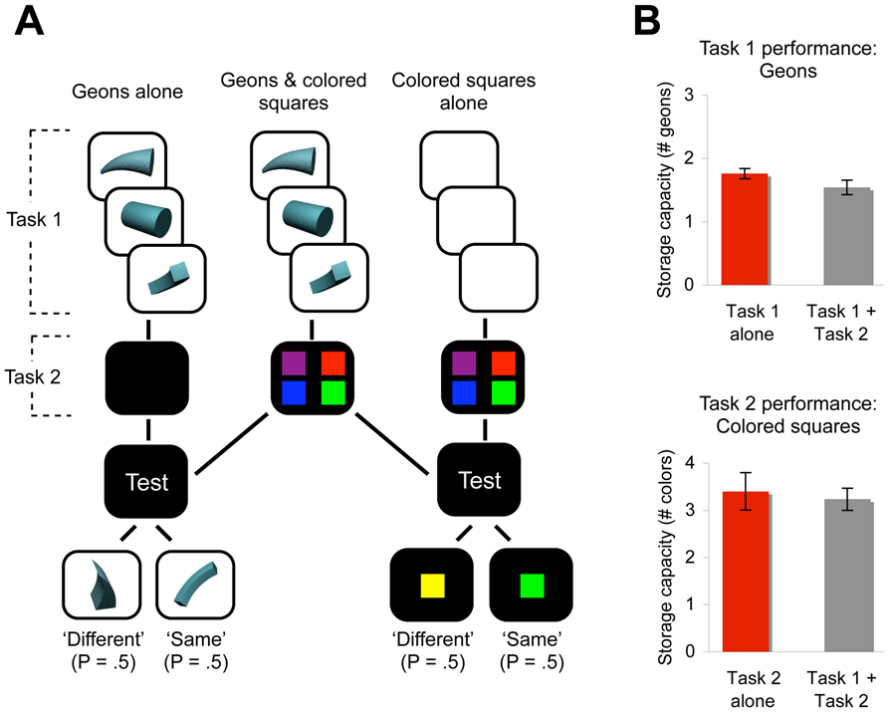
Design and Results from Experiment 2. (A) Flow chart of events for the dual-task working memory method. In the first memory task, observers attempted to remember 0–3 3D geons, each presented for 500 ms and followed by a 500-ms inter-stimulus interval. In the second memory task, observers attempted to remember 0, 2, 4, or 6 colored squares presented in a single, 500-ms display. After a 1000-ms delay interval, a test item appeared which consisted of a geon (50% of trials) or a colored square (50% of trials). Observers indicated whether the test item had been present in the trial, which was true on 50% of the trials. (B) The number of geons remembered from the first memory task and the number of colored squares remembered from the second memory task. Red bars indicate trials in which the memory tasks were performed alone and grey bars indicate trials in which the memory tasks were performed concurrently with the most difficult trials from the other memory task. Error bars represent standard error.

Accordingly, Experiment 2 used a dual-task method to examine whether view-dependent and view-invariant representations compete for limited visual working memory storage resources. In the first memory task, observers attempted to remember 0–3 3D geons presented from different viewpoints in the sample and test arrays. Thus, to succeed in this first memory task, observers would need to sustain view-invariant representations of the geons. In the second memory task, observers attempted to remember 0, 2, 4, or 6 colored squares presented in a view/display (Figure 3A). Colored squares were used for two reasons. First, they are arguably the most simple, suprathreshold feature that can be sustained within a view-dependent representation [13], [16]. Second, they have been used extensively to study properties of visual working memory [13]–[16], which creates a link between the present findings and those previous studies.

Single-system and two-system models make contrasting predictions about whether the two memory tasks will compete for visual working memory resources. Single-system models predict that representations of 3D objects and colored squares will be sustained in the same visual working memory system. Thus, the two memory tasks should compete for the same 3–4 storage slots of visual working memory, making it significantly more difficult to perform the two tasks concurrently compared to alone. In contrast, the two-system model predicts that representations of 3D objects and colored squares can be sustained in separate visual working memory systems. Thus, observers should use both memory systems in parallel to perform the tasks, allowing observers to perform both memory tasks concurrently nearly as well as they can perform the tasks alone.

Results provide strong support for the two-system model: Observers remembered 1.76 geons when performing the first memory task alone and 1.54 geons when performing the task concurrently with the most difficult trials from the second task, and 3.40 colored squares when performing the second memory task alone and 3.23 colored squares when performing the task concurrently with the most difficult trials from the first task (Figure 3B; Table 2). Thus, observers performed both memory tasks concurrently nearly as well as they performed the tasks alone. Strikingly, the total number of items that could be remembered across the two memory tasks was nearly identical whether the tasks were performed separately or concurrently, *F*(1,9) = 0.81, *P* = .39. Further, the magnitude of the small dual-task cost, 0.39 items, was no greater than the 0.60 – 0.80-item cost observed in previous dual-task experiments that placed high loads on two separate working memory systems concurrently (i.e., visual and verbal working memory) [24], and thus, presumably reflects demands on a more general component of working memory [25]. Although a single system is commonly accepted, the independence between these visual working memory tasks indicates that visual working memory can be divided into two separate, specialized systems for sustaining non-spatial object information, as opposed to a single system, as is commonly thought.

**Table 2:** Proportions of Hits and False Alarms (FAs) for Experiment 2 (Hits/FAs).

	Stimuli Type	0 colored squares	2 colored squares	4 colored squares	6 colored squares
**0 geons**	**geons**	N/A	N/A	N/A	N/A
	**colored squares**	N/A	.96/.03	.94/.17	.89/.28
**1 geon**	**geons**	.97/.13	.82/.17	.97/.17	.80/.17
	**colored squares**	N/A	.98/.03	.90/.23	.77/.45
**2 geons**	**geons**	.93/.21	.92/.23	.77/.30	.83/.12
	**colored squares**	N/A	.98/.05	.85/.22	.92/.30
**3 geons**	**geons**	.83/.13	.80/.18	.88/.42	.75/.20
	**colored squares**	N/A	.97/.08	.92/.15	.85/.28

Includes proportion of Hits (responding *different* on change trials) and False Alarms (responding *different* on same trials) for each of the conditions.

These results cannot be explained by appealing to the spatial working memory system because there is no evidence that spatial working memory can represent detailed object form and surface feature information, which was needed to succeed in the memory tasks used in this experiment. It will be interesting for future studies to investigate how the view-invariant and view-dependent working memory systems interact with spatial working memory to produce an integrated representation of the visual environment.

### Experiment 3

The near lack of dual-task interference in Experiment 2 could be explained by the use of separate, specialized memory systems for sustaining sequentially and simultaneously presented information rather than separate, specialized systems for view-invariant and view-dependent representation. This alternative hypothesis predicts that geons presented sequentially and simultaneously will not compete for working memory storage resources when the objects from both memory tasks need to be sustained in a view-invariant format. To test this prediction, the number of view-invariant representations required during the dual-task method was manipulated by varying whether or not the geons in each task needed to be recognized across a viewpoint change.

**Figure 4:**
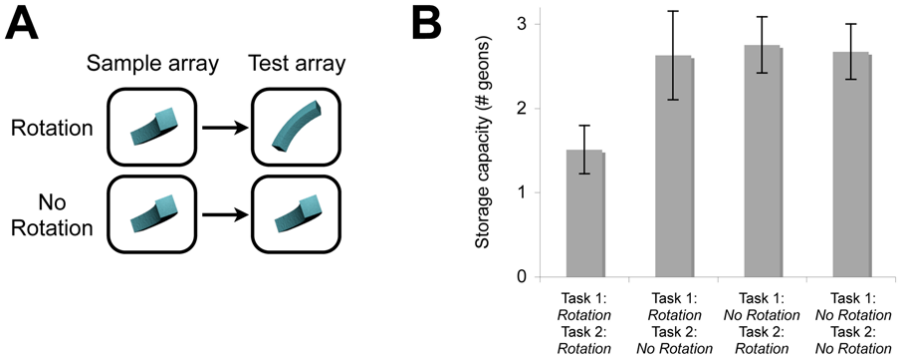
Sample Stimuli and Results from Experiment 3. (A) Example of a geon presented from the same viewpoint and from a different viewpoint in the sample array and test array. (B) The number of geons successfully remembered in each condition when the geons in both memory tasks, the first memory task, the second memory task, or neither memory task needed to be recognized across a viewpoint change. Error bars represent standard error.

Observers (*n* = 10) attempted to remember three sequentially presented geons in the first memory task and four simultaneously presented geons in the second memory task. In separate conditions, the geons in the first memory task, the second memory task, both memory tasks, or neither memory task needed to be recognized across a viewpoint change (Figure 4A). Results revealed significant interference between the tasks when the geons from both memory tasks needed to be recognized across a viewpoint change. Observers remembered only 1.51 geons when the geons in both tasks needed to be recognized across a viewpoint change (Figure 4B; Table 3); however, when a subset of the geons did not need to be recognized across a viewpoint change, and could therefore be sustained within view-dependent representations, the number of geons that could be remembered successfully increased by 76%, *F*(1,9) = 11.53, *P* = .008. Thus, as in Experiment 1, observers sustained 1–2 view-invariant representations, while the remaining objects were sustained within view-specific representations. These data rule out the alternative explanation that separate, specialized memory systems sustain sequentially and simultaneously presented information. Further, the fact that performance was nearly identical when a subset of the geons compared to none of the geons needed to be recognized across a viewpoint change (*P*s>.80) is inconsistent with single-system models in which view-invariant representations require more storage resources from a single visual working memory system than view-dependent representations.

**Table 3:** Proportions of Hits and False Alarms (FAs) for Experiment 3 (Hits/FAs).

Condition	Hits/FAs
**Task 1: Different viewpoints**	.66/.35
**Task 2: Different viewpoints**	.51/.38
**Task 1: Same viewpoints**	.76/.31
**Task 2: Same viewpoints**	.45/.12
**Task 1: Different viewpoints**	.77/.38
**Task 2: Same viewpoints**	.53/.20
**Task 1: Same viewpoints**	.71/.14
**Task 2: Different viewpoints**	.56/.30

Includes proportion of Hits (responding *different* on change trials) and False Alarms (responding *different* on same trials) for each of the conditions.

Observers sustained 3–4 items in view-dependent memory in Experiment 2 but only about 1 item in view-dependent memory in Experiment 3. What accounts for this difference in the storage capacities across experiments? Many studies have shown that fewer objects can be sustained with high fidelity in visual working memory as the objects become more complex [14], [16]. Thus, the storage capacity of visual working memory should have been lower for the geons in Experiment 3 than for the color values in Experiment 2 because the two-dimensional contours of a three-dimensional geon are more complex than a color value.

The data from Experiment 1 indicate that between 100 ms and 1,000 ms, view-dependent memory accumulated between 0.78 and 1.36 item's worth of information from a scene of 3D objects (see Materials and Methods). In Experiment 3, view-dependent memory accumulated 1.12 item's worth of information when four geons were presented in a 500-ms display (see Materials and Methods). Thus, observers sustained similar amounts of view-dependent information in Experiment 1 (when all memory items appeared in one display) and Experiment 3 (when all memory items appeared across four displays). Likewise, observers sustained nearly identical numbers of view-invariant representations in Experiments 1 and 3. This pattern cannot be explained by appealing to state-dependent memory stores or limitations in retrieval processes because these alternative explanations predict higher performance when the memory items are presented across multiple displays compared to on the same display.

Finally, analyses of error patterns show that observers were more likely to incorrectly believe that a geon was replaced with a new geon (i.e., to false alarm) when the geons were rotated versus not rotated in the test arrays, both in the first memory task, *F*(1,9) = 5.55, *P* = .04, and in the second memory task, *F*(1,9) = 9.88, *P* = .01. This same pattern was observed in Experiment 1. It suggests that view-dependent representations of the sample array mismatched with the sensory properties of the rotated objects in the test array, creating the perception that a geon was replaced with a new geon.

### Experiment 4

Another alternative explanation is that despite performing an articulatory suppression task throughout each trial, observers nonetheless sustained information about a subset of the objects using verbal working memory. To explore this possibility in a different way, in Experiment 4 I manipulated the type of object change that could occur in each memory task.

In one condition, objects were replaced by a different object from the same basic-level category (e.g., a guitar replaced by a guitar), and in a second condition, objects were replaced by an object from a different basic-level category (e.g., a guitar replaced by an anchor) [26]. As in Experiments 1–3, observers performed the articulatory suppression task throughout each trial. If observers remember objects using verbal working memory, then performance should be substantially lower in the first condition because a more detailed verbal representation is needed to distinguish between two items that are visually distinct but are in the same basic-level category compared to two items from different basic-level categories (e.g., remembering the word “guitar” cannot distinguish between two visually distinct guitars). Performance was nearly identical across conditions, *F*(1,9) = 0.75, *P* = .41 (Table 4), indicating that verbal working memory does not sustain a significant portion of information in this dual-task method. Further, this final experiment suggests that both view-dependent and view-invariant representations contain considerable object detail, containing at least enough visual details to distinguish specific items from the same basic-level category.

**Table 4:** Proportions of Hits and False Alarms (FAs) for Experiment 4 (Hits/FAs).

Condition	Hits/FAs
**Task 1: Different basic-level category**	.86/.28
**Task 2: Different basic-level category**	.63/.06
**Task 1: Same basic-level category**	.79/.28
**Task 2: Same basic-level category**	.56/.06
**Task 1: Different basic-level category**	.90/.31
**Task 2: Same basic-level category**	.56/.11
**Task 1: Same basic-level category**	.80/.29
**Task 2: Different basic-level category**	.66/.08

Includes proportion of Hits (responding *different* on change trials) and False Alarms (responding *different* on same trials) for each of the conditions.

## Discussion

This study provides evidence that visual working memory can be divided into two separate, specialized systems for sustaining view-dependent and view-invariant representations. When observers were asked to remember scenes of 3D objects, the storage capacity of visual working memory was subject to two independent limits, one on view-dependent sensory representation and one on view-invariant object representation. This effect was not due to limitations in processes other than working memory storage, such as those used to perceive the stimuli, encode the information into memory, or retrieve the information from memory. Nor was the effect due to the storage of information in verbal working memory or spatial working memory.

Many previous studies have examined the nature of visual working memory for 2D objects, and the results from these studies have traditionally been interpreted as reflecting properties of a single memory system [13]–[16]. However, such 2D stimuli can be successfully remembered using both view-dependent sensory representations and view-invariant representations of individual items, because the items in the sample and test arrays could have been compared on the basis of either the sensory features of the displays or the identities of the individual items. As a result, previous studies of visual working memory [e.g.], [ 13]–[16] may have inadvertently elicited representations from two different memory systems.

The stimuli used in this study included the most simple, suprathreshold units that can be sustained in a view-dependent representation (colored squares) and the most simple, suprathreshold units that can be used to study 3D object representation (3D geons). These results therefore characterize the storage capacities of both view-dependent sensory representation and 3D object representation. These storage capacity estimates may be of considerable value to the many fields in cognitive science that use capacity limits on information storage to understand the nature of visual cognition.

It will be interesting for future research to examine how the view-dependent and view-invariant memory systems support the wide range of tasks that depend on visual working memory, including object recognition, saccadic memory, scene perception, navigation and imagination. View-invariant memory may play a specialized role in tasks that require identifying individual objects, whereas view-dependent memory may play a specialized role in navigation tasks where view-specific representations characterize particular locations in the environment.

These results dovetail with two other lines of research. First, behavioral studies show that ants, bees, wasps, rodents and humans navigate the visual environment on the basis of view-specific representations of the scene [1]–[10]. These results have been taken as evidence for a phylogenetically primitive module which uses the sensory features of the surroundings to determine current location.

Second, neurophysiological studies show that visual working memory tasks activate neural substrates from both early (V1-V4) and late (e.g., lateral occipital complex) levels of the visual hierarchy [27]–[30]. Although researchers typically interpret these varying patterns of activation as reflecting substrates of a single memory system, they may alternatively reflect the activation of the view-dependent and view-invariant memory systems described here. Representations in early levels of the hierarchy are pixel-like pictorial representations of the scene, akin to photographs. Visual working memory representations sustained within these early levels of the hierarchy may therefore be view-dependent and could support visual processes that operate over view-dependent information [1]–[10]. In contrast, later levels of the visual hierarchy support object-recognition mechanisms that represent perceived 3D shape, but not low-level image features [31], [32]. Visual working memory representations sustained within later levels of the hierarchy may therefore be view-invariant and could support visual processes that operate over representations of individual objects.

These results shed light on the processes subserving the temporary storage of visual information and on their relation to information storage in other animals. Moreover, they place constraints on accounts of the processes that guide visual representation in humans.

## Materials and Methods

### 

#### Participants

Ten new participants participated in each experiment. All gave informed consent. This research was approved by the University of Southern California Human Subjects Committee.

### Experiment 1

#### Procedure

The participants (male: 0; female: 10) were between the ages of 18 and 21 (*M* = 19.7, SD = 0.95). Each trial began with a 1000-ms presentation of two randomly selected letters and participants were required to repeat those letters continuously and out loud until the end of the trial. The offset of these letters was followed by a 1000-ms presentation of a black screen, followed by the 500-ms presentation of a white box subtending 9.5° (height)×15° (width) at the center of the screen. The sample array then appeared within the white box, which consisted of five geon objects equally spaced around an invisible circle with a radius of 3.25°. On average, each geon subtended 2.5° (height)×2.5° (width). In separate conditions, the sample array was displayed for 100 ms, 1,000 ms, 2,000 ms, and 3,000 ms. After a 1,000-ms delay interval, the test array appeared and participants indicated whether or not the geons in the sample array and test array were the same or whether one of the geons had been replaced with a new geon. The test array remained visible until participants made a response. On 50% of the trials, the arrays contained the same geons; on the other 50% of the trials, one geon was replaced with a new geon that was different from all of the geons in the sample array. In the ‘no rotation’ conditions, the geons were presented from the same viewpoints in the sample array and test array. In the ‘rotation’ conditions, the geons were presented from different viewpoints in the arrays, rotated 45° or 90° (randomly selected, see Fig. 1 for geon stimuli set). Participants received 50 trials for each unique combination of sample array display time (100 ms, 1,000 ms, 2,000 ms, 3,000 ms) and rotation condition (rotation, no rotation). Participants were instructed before each condition whether the objects would be presented from the same viewpoints or from different viewpoints in the sample array and test array. Each condition was preceded by four practice trials. For the statistical analyses, negative capacity estimate values were replaced with capacity estimate values of 0 because it is not possible for an observer to remember a negative number of items in a condition. Nearly identical statistical patterns obtained whether or not the data were transformed in this manner.

A view-invariant representation could be used to detect an object change in both the ‘rotation’ and ‘no rotation’ conditions; in contrast, a view-dependent representation could be used to detect an object change in the ‘no rotation’ conditions only. Thus, to provide estimates of the number of item's worth of view-dependent information that observers accumulated from the sample arrays for each sample array display time, I computed the differences in storage capacities between the ‘rotation’ and ‘no rotation’ conditions.

See Table 1 for full results including proportions of Hits and False Alarms for all conditions.

### Experiment 2

#### Procedure

The participants (male: 6; female: 4) were between the ages of 19 and 29 (*M* = 21.4, SD = 2.84). Each trial began with a 1000-ms presentation of two randomly selected letters and participants were required to repeat those letters continuously and out loud until the end of the trial. The offset of these letters was followed by a 500-ms presentation of a black screen, followed by a 500-ms presentation of a white box subtending 3.5° (height)×5.5° (width) at the center of the screen. For the first memory task, 0–3 geons were then presented in sequence at the center of the screen. Each geon was presented for 500 ms, followed by a 500-ms inter-stimulus interval. The last geon in the sequence was followed by a 500-ms black screen, followed by the 500-ms presentation of the stimuli from the second memory task, which consisted of 0, 2, 4, or 6 colored squares (red, orange, yellow, green, blue, white, purple) on a black background. For the 2-object arrays, the objects (1.75°×2°) were presented on the horizontal midline, offset 3.5° from the center of the screen. For the 4-object arrays, the objects were presented equidistant from the middle of the screen in four quadrants, offset 1.5° from the horizontal midline and 3.5° from the vertical midline. For the 6-object arrays, 2 objects were presented on the horizontal midline, offset 3.5° from the middle of the screen, and the remaining 4 objects were offset 3° above and below those objects. After a 300-ms delay interval, there was a 500-ms presentation of the word “test,” followed by a 200-ms delay and then the test item. The test item consisted of a single geon (50% of trials) or colored object (50% of trials) presented at the center of the screen. Participants indicated whether the test item had been present in the trial, which was true on 50% of the trials. Participants received 24 trials for each unique set size combination of geons (0, 1, 2, 3) and colored squares (0, 2, 4, 6).

The storage capacity values reported in the main text were computed by averaging the capacity estimate values from the trials that tested memory for the two largest set sizes (i.e., trials testing memory for 2 & 3 geons in Task 1; trials testing memory for 4 & 6 colored squares in Task 2).

See Table 2 for full results including proportions of Hits and False Alarms for all conditions.

### Experiment 3

#### Procedure

The participants (male: 1; female: 9) were between the ages of 19 and 27 (*M* = 20.6, SD = 2.41). Each trial began with a 1000-ms presentation of two randomly selected letters and participants were required to repeat those letters continuously and out loud until the end of the trial. The offset of these letters was followed by a 1000-ms presentation of a white box subtending 9° (height)×11° (width) at the center of the screen. For the first memory task, the geons were then presented in sequence at the center of the screen. Each geon was presented for 500 ms, followed by a 500-ms inter-stimulus interval. The last geon in the sequence was followed by a 500-ms delay interval, followed by the presentation of the stimuli from the second memory task, which consisted of four geons in a single 500-ms display. The geons were presented equidistant from the middle of the screen in four quadrants, offset 1.5° from the horizontal midline and 2.5° from the vertical midline. After a 900-ms delay interval the test array was presented. The test array consisted of a single geon from the first memory task presented at the center of the screen (50% of trials) or a display of four geons from the second memory task (50% of trials). Participants indicated whether the test geon(s) had been present in the trial, which was true on 50% of the trials. In separate conditions, the geons were presented from different viewpoints in the sample and test arrays in the first memory task, in the second memory task, in both memory tasks, or in neither memory task. Participants received 64 trials in each condition.

To compute the number of item's worth of view-dependent information accumulated from the 500-ms display in the second memory task, I computed the difference between the storage capacities from the condition in which the objects in both memory tasks were rotated between the sample and test arrays and the condition in which only the objects in the first memory task were rotated between the sample and test arrays.

See Table 3 for full results including proportions of Hits and False Alarms for all conditions.

### Experiment 4

#### Procedure

The participants (male: 3; female: 7) were between the ages of 18 and 22 (*M* = 19.9, SD = 1.52). The methods used in Experiment 4 were identical to those used in Experiment 3 except in two ways. First, the geons were replaced with objects from the Hemera Object Database and Google Image Search, courtesy of Tim Brady, Massachusetts Institute of Technology, http://cvcl.mit.edu/MM/download.html. Second, the type of object change that could occur in each memory task was manipulated across the conditions. On change trials, an object could either be replaced with a different object from the same basic-level category (e.g., a guitar replaced by a different guitar) or with an object from a different basic-level category (e.g., a guitar replaced by an anchor). Participants received 64 trials in each condition.

See Table 4 for full results including proportions of Hits and False Alarms for all conditions.
